# Unblocking Barriers of Access to Hepatitis C Treatment in China: Lessons Learned from Tianjin

**DOI:** 10.5334/aogh.2763

**Published:** 2020-04-06

**Authors:** Peiwen Zhang, Ran Guo, Jun Lian, Mengjia Zhi, Chengzhen Lu, Weishen Wu, Lihong Wang, Polin Chan, Zhongdan Chen, Jing Sun

**Affiliations:** 1 School of Public Health, Chinese Academy of Medical Sciences and Peking Union Medical College 5 Dongdansantiao, Beijing, CH; 2 Department of Chronic Hepatitis, Tianjin No 2 People’s Hospital, Tianjin, CH; 3 Department of Communicable Diseases Control, Tianjin Center for Disease Control and Prevention, Tianjin, CH; 4 Tianjin Healthcare Security Administration and Settlement Center, Tianjin, CH; 5 HIV, Hepatitis and STI Unit, Division of Communicable Diseases, World Health Organization Western Pacific Regional Office, Manila, PH; 6 Hepatitis/TB/HIV/STI, World Health Organization, Office of the WHO Representative in China, Beijing, CH

## Abstract

**Background:**

The high price is a critical barrier of access to new direct-anting-antiviral (DAA) therapies for hepatitis C for both the patients and the society. Many countries continue to face the challenge of financing such expensive medicines. Such examples include both high-income and middle-income countries. Existing evidence about the efforts of China to address this challenge is limited. To our knowledge, this is the first detailed description of a novel financing model and comprehensive analysis of its impact on patient financial burden of hepatitis C treatment in China.

**Objective:**

To examine the evolution of approaches to navigating patients’ barriers of access to DAA-based treatment of hepatitis C in Tianjin City, China.

**Methods:**

Review of publicly available literature, including published and grey literature. Conduct on-site data extraction and key informant interview. The patient financial burden of hepatitis C treatment was analyzed. The financial burden of hepatitis C patients with different treatment models and health insurance financing models was measured by calculating the number of annual income to cover patient out-of-pocket (OOP) expenditure for the standard treatment course accordingly.

**Findings:**

Tianjin is the first area to pilot a capitated provider payment program for the treatment of hepatitis C. Through which, the retirees and employees spend 0.7 and 1.0 months of their salary, and residents spend 5.6-6.8 months of their salary for the treatment, the financial burden of patients were much relieved. By the end of March 2019, the first-year pilot program had 876 hepatitis C patients registered the new insurance coverage and treated in Tianjin.

**Conclusions:**

The study showed that the financial barriers of access to new hepatitis C treatment for patients could be unblocked with government commitment and novel financing models. International experiences demonstrated that centralized bulk procurement is a good leverage for price negotiation, primarily when using innovative payment approaches. To replicate the initial success of Tianjin, continued efforts are needed for stronger strategic price negotiation, preferably at central level. The case of Tianjin brings implications to the other areas of China and even other developing countries that government commitment, novel financing model and pooled procurement are critical elements of stronger purchasing power and a better secure of treatment.

## Introduction

The global number of viral hepatitis-related deaths was comparable with the number of deaths from tuberculosis, but exceeded the number of deaths from HIV in 2015. Seventy-one million people were estimated to be living with hepatitis C viral (HCV) worldwide, and an estimated 399,000 persons died from the consequences of HCV infection in 2015 [1]. The World Health Organization (WHO) called for the elimination of viral hepatitis as a public health threat by 2030. Specific global targets were set on prevention and control of viral hepatitis, such as new infections and deaths to be reduced by 90% and 65% respectively [1]. The Western Pacific Region bears one-third of the world’s deaths caused by hepatitis, translating to more than 1,200 deaths every day. The estimated number of population living with chronic hepatitis C in 2016 in this region was 14 million. The epidemic of viral hepatitis takes a heavy toll on lives and health systems for countries [2]. China has a substantial burden of chronic viral hepatitis. The estimated HCV prevalence is 0.43%, which is not at a high level by comparison to surrounding countries [3]. The absolute number (7.6 million) is substantial [4,5]. New cases of hepatitis C reported annually exceed 200,000 in the most recent four years [6].

Interferon-based regimens have been the sole therapies of HCV for decades, but are associated with severe adverse events and low virus clearance rate. The recent discovery of oral direct-acting antivirals (DAAs), for their high cure rate and demonstrated safety, heralded a revolution and changed the landscape of hepatitis C treatment and prevention. The current standard treatment recommended by the World Health Organization was presented in Annex 1. However, high prices of these new therapies became barriers for patients to access treatment. Many countries continue to face the challenge of financing such expensive medicines. To reduce the disease burden through HCV cure, countries adopted different approaches tailored for their specific context. Such examples include both the developed and the developing countries like Australia and Brazil through lump-sum remuneration, and Productive Development Partnerships (PDPs) approaches [7,8].

In responding to the global vision of eliminating hepatitis C as a public health threat, the Chinese government also took several actions. The Prevention and Treatment Guideline for Hepatitis C was updated to incorporate the latest DAAs as part of standards of treatment for hepatitis C in 2015 [5]. In 2017, the National Viral Hepatitis Prevention and Control Plan (2017–2020) was developed and released [9]. The National Diagnosis Standard for Hepatitis C was updated in 2018 [10], which for the first time presented the distribution of HCV genotypes in China to support better clinical decision making. In 2016, DAAs were included in the new priority review channel for regulatory approvals of novel medicines, which was part of the regulatory reforms. The first oral DAA, daclatasvir-asunaprevir, which officially enabled China to enter a new era of hepatitis C treatment, was marketed in April 2017. Several other DAAs followed this process, including the first locally developed DAA, danoprevir, in 2018. By the end of March 2019, nine DAAs were registered, and three more were undergoing clinical trials in China (Annex 2) [11].

In spite of various efforts, treatment rate of hepatitis C is still low in China, estimated at less than 1.3% [12]. Apart from low awareness of the disease, patient affordability is one of the most critical reasons for the low treatment. Access of DAAs remains limited due to the very high retail prices, ranging between US$ 10,000–17,600 per standard treatment course in the private sector in 2018.^1^

The approach to unblock the financing barriers to these high-priced medicines and to ensure the reduction of out-of-pocket (OOP) expenses in China was to cover these medicines by the health insurance program. In October 2018, the National Health Commission (NHC) announced the inclusion of a pan-genotypic DAA combination, sofosbuvir/velpatasvir in the National Essential Medicines List [13]. However, listed as essential medicines does not mean it can be publicly funded in China, as patients can only get access to publicly funded medicines through a health insurance program. Being listed by a health insurance program is a prerequisite of getting access by major patients. Different government agencies administer the selections of essential medicines and reimbursed medicines with different processes, and by different expert committees. NHC is in charge of listing essential medicines, and the newly established National Healthcare Security Administration (NHSA) takes care of listing reimbursed medicines. Until the end of April 2019, none of the nine marketed DAAs was listed by NHSA yet, including the one listed by NHC as national essential medicines.

With the increasing availability of new DAAs in the Chinese market, several local health insurance programs piloted initiatives to improve patient’s financial accessibility. Zhejiang province pioneered to have sofosbuvir and sofosbuvir/velpatasvir covered by the provincial catastrophic health insurance program [14]. Tianjin, Chengdu and Changchun city, Jiangsu, Shandong, Jilin and Anhui provinces followed the example of Zhejiang province. Most of the local health insurance programs negotiated prices of DAAs, and set a fixed proportion of co-payment with the patient, with insurance reimbursement rates ranging between 40–90%. Patients have to pay a deductible of nearly US$ 3,000 and another 10–20% of the total expenditure OOP before getting insurance reimbursement.

Tianjin is a city in the east of China, it is one of the four municipalities directly under the central government. It is an economic centre in the Bohai Bay, the largest open coastal city in the north of China, with 15.60 million permanent residents [15]. The 2010 point of prevalence survey of hepatitis C showed that the prevalence rate among the registered permanent residents in Tianjin was 24.10 per 100,000 (132/547,782) [16]. A 2013 study found that the mortality rate of hepatitis C–related cirrhosis and liver cancer was 0.6 per 100,000 [17]. Data from the Notifiable Communicable Diseases Reporting System of Tianjin showed that a total of 8,421 new cases of hepatitis C were reported during 2004–2018 (914 in 2018). The notification rate of hepatitis C in 100,000 permanent residents ranged from the highest of 7.03 in 2006, dropped steadily to the lowest of 2.95 in 2012. Notifications increased after 2012 and reached 5.86 per 100,000 permanent residents in 2018 [18,19,20]. According to the latest national data in 2016, the incidence of hepatitis C in Tianjin was 3.57 per 10,000 population, far below the national level (15.09/10,000 population) [20]. Among the several local health insurance programs who negotiated the prices of DAAs, Tianjin was the first one to pilot a capitated provider payment for hepatitis C treatment with defined clinical pathway and DAAs included in 2018. The insurance program prospectively set the per-patient price to be paid to hospitals in a lump sum rather than retrospective fee-for-service (FFS) provider payment. The policy objectives were to improve patient’s affordability of DAA treatment further, increase cost awareness of healthcare providers and efficiency of the insurance fund [21]. Comparing the international exposure of government efforts in improving the affordability of DAA treatment, there is limited information about the detailed financing models and patient affordability in China. This study examines the evolution of approaches to navigate the financial barriers of patients to access to DAAs for HCV in Tianjin city, China. To our knowledge, this is the first study to conduct a detailed description of a novel health insurance financing model implemented in developing countries of DAA treatment for hepatitis C. This is also the first study to provide a comprehensive analysis of the patient financial burden for hepatitis C treatment.

## Methods

We reviewed published literature and a substantial body of evidence available in grey literature, including landmark or highly regarded reports, work suggested by peers and documents of government and international organizations. The study extracted on-site data from the database of the Tianjin Healthcare Security Management and Settlement Centre and Hepatitis C Patient Assistance Programme initiated by the China Primary Health Care Foundation. This study also drew on insights of key informants from relevant central (Beijing) and local government agencies in charge of health administration, healthcare security administration, disease control and prevention; and specialty hospitals in Tianjin. Other stakeholders, including international organizations, non-governmental organizations and pharmaceutical companies also contributed to the study (for a list of key informants, see Annex 3). The financial burden of hepatitis C patients with different treatment models and health insurance financing models was measured by calculating the number of annual income to cover patient OOP expenditure for the standard treatment course under different financing models in three stages: fee-for-service provider payment for interferon-based treatment before 2014; capitated provider payment for interferon-based treatment during 2014 to 2017; and capitated provider payment for DAA-based treatment with defined clinical pathway since 2018.

## Results

### Fee-for-service provider payment for interferon-based treatment before 2014

**Figure 1:**
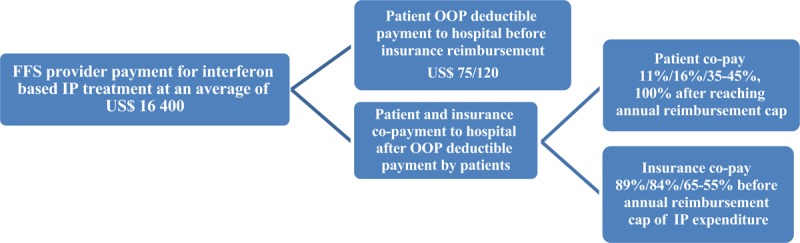
Fee-for-service provider payment for interferon based treatment before 2014. *Notes:* FFS = fee-for-service; IP = inpatient; OOP = out-of-pocket; deductible = patient OOP payment to hospital before insurance reimbursement.

Before DAAs were marketed in China, interferon therapy was the only regimen recommended for the treatment of hepatitis C under the coverage of Tianjin Basic Health Insurance. The standards for treatment were a dose of 180μg interferon, one subcutaneous injection per week for 6–12 months, and usually combined with oral ribavirin. Nearly all patients relapsed after treatment and required re-treatment [18]. Repeated injections lead to poor compliance and tolerance, and involved additional treatment for adverse drug reactions (ADRs). Patients have to pay a deductible amount of US$75 (residents) and US$120 (employees) OOP before getting reimbursed, and there is an annual reimbursement cap (US$26,900 for the residents and US$52,240 for the employees). Most hepatitis C patients chose comparatively expensive inpatient care as a way to access to a better benefit package rather than outpatient care (deductible at US$75 for the residents and US$90 for the employees, annual reimbursement cap up to US$450 for the residents and US$820 for the employees) [22,23]. The actual annual expenditure of frequent hospitalization was around US$16,400, including charges for bed and inpatient care, diagnosis and tests, interferon, ribavirin and other medicines to manage ADRs [24]. After patient payment of a deductible amount, 11%, 16% and 35–45% of the expenditure were paid OOP by the retirees, the employees and the residents (higher premium contribution getting lower OOP). The patient OOP payment accounted for 2.2 and 3.2 months of the retirees’ and employees’ salary, and 17.6–22.6 months of the residents’ disposable income (Figure 1 and Table 1). The insurance-designated hospitals were co-paid by insurance and patients for the actual expenditure with the retrospective FFS method.

**Table 1:** Benefit package, expenditure, payment and cost-sharing of hepatitis C treatment in Tianjin in three phases.

	Before 2014	2014–2018		After 2018					

Insurance reimbursementpolicies/Population	Employees/Retirees^1^	Residents (low to high premium contribution)^2^	Employees/Retirees^1^	Residents (low to high premium contribution)^2^	Employees/Retirees^3^	Residents (low to high premium contribution)^4^	Patients in financial hardship ^5^	Low-income requiring more than 3-month treatment^6^	

								Employees/Retirees	Residents (low to high premium contribution)

Benefit package	Inpatient		Inpatient (day-care-ward)		Outpatient (specialty)		PAP + common outpatient^7^	Specialty outpatient^8^ + PAP + common outpatient^7^	
Medicines & services covered	Interferon + ribavirin + ADR treatment + tests	Interferon + ribavirin + ADR treatment+ tests	All DAAs marketed in China + interferon + ribavirin + ADR treatment + tests		Originator sofosbuvir	Originator sofosbuvir	Medicines & services covered	Interferon + ribavirin + ADR treatment + tests	Interferon + ribavirin +ADR treatment + tests
Capitated amount per standard treatment course (US$)	16,400^9^	11,300^10^	6,045^11^		PAP covered^12^ + tests & medications for ADRs	6,045^11^ + PAP covered^13^ + tests & medications for ADRs	Cost per treatment course (US$)	16,400^9^	11,300^10^
Patient OOP to hospitals before insurance reimbursement (US$)	120	75	0		194	75	NA+75^7^	194^8^ + NA + 120/90^7^	75^8^ + NA + 75^7^
Insurance reimbursement rate after initial patient OOP (%)	84/89	55–65	85/90	55–65	85/90	45–55	NA/50^7^	85/90^8^/NA/55/95^7^	45–55^8^/NA/50^7^
Max insurance payment to hospitals (US$)	13,838/14,652	8,979–10,611	9,605/10,170	6,215–7,345	4,973/5,266	2,687–3,284	Up to 450^7^	4,973/5,266+up to 820–1,500^7^	2,687/2,985/3,284 + up to 450^7^
Max patient OOP to hospitals (US$)	2,562/1,784	7,421–5,789	1,695/1,130	5,085–3,955	1,072/780	3,358–2,761	Tests & ADR medications	1,072/780 + tests & ADR medications	3,358/3,060/2,761 + tests & ADR medications
Max patient OOP rate (%)	16/11	45–35	15/10	45–35	18/13	55–45	NA	18/13 plus^14^	55/50/45plus^14^
No. of per capita monthly income for max patient OOP to hospitals	3.2/2.2^15^	22.6–7.6^16^	1.6/1.1^15^	12.5–9.7^16^	1.0/0.7^15^	6.8–5.6^16^	NA	1.0/0.7^15^ plus^14^	6.8–5.6^16^ plus^14^

*Notes:* PAP = patient-assistance program; OOP = out of pocket; DAA = direct-acting antiviral; ADR = adverse drug reaction. Max insurance settlement with hospitals and max patient OOP were estimated based on the capitated amount per standard treatment course. Benefit package, patient OOP before getting insurance reimbursed, and insurance co-pay (%) were obtained from the key informants of Tianjin Healthcare Security Administration and Tianjin Healthcare Security Management and Settlement Center. Comprehensive insurance policies about retirees/employees and residents of Tianjin can be accessed from the website of Tianjin Human Resource and Social Security Bureau as follows: (http://hrss.tj.gov.cn/ecdomain/framework/tj/ckoocoapccolbbogkjpnifpkdgnfeahc/clbhpndaccolbbogkjpnifpkdgnfeahc.do?isfloat=1&fileid=20081114155807953&moduleIDPage=clbhpndaccolbbogkjpnifpkdgnfeahc&siteIDPage=tj&pageID=ckoocoapccolbbogkjpnifpkdgnfeahc, in Chinese, accessed March 31, 2019) and (http://hrss.tj.gov.cn/ecdomain/framework/tj/ckoocoapccolbbogkjpnifpkdgnfeahc/clbhpndaccolbbogkjpnifpkdgnfeahc.do?isfloat=1&fileid=20130428083007295&moduleIDPage=clbhpndaccolbbogkjpnifpkdgnfeahc&siteIDPage=tj&pageID=ckoocoapccolbbogkjpnifpkdgnfeahc, in Chinese, accessed March 31, 2019). ^1^ Employee beneficiaries of Tianjin Basic Health Insurance. ^2^ Residents beneficiaries of Tianjin Basic Health Insurance. ^3^ Employee beneficiaries of Tianjin Basic Health Insurance qualified at least three consecutive years. ^4^ Resident beneficiaries of Tianjin Basic Health Insurance qualified at least three consecutive years. ^5^ Patients in financial hardship as certified by the Tianjin Civil Affairs Bureau, and receive a minimal living allowance during the past consecutive 12 months. ^6^ Annual family (immediate family, defined as family members including parents, children, husband or wife) income ≤ 2.5 times of the annual family healthcare expenditures, information obtained from the key informants of PAP. ^7^ Costs for tests & other necessary medications for ADRs can be reimbursed by insurance following the common outpatient benefit package. ^8^ The first three-month treatment with originator sofosbuvir of low-income patients before applying for free originator sofosbuvir can be reimbursed by insurance following the specialty outpatient care benefit package under the capitated payment program. ^9^ Average costs per treatment course (CNY 110,000/48 weeks) before 2014, as reported by Ni S, Wang L. Chronic hepatitis C treatment cost is reduced in Tianjin. Tianjin Social Insurance, 2014(3):30–31. ^10^ The capitated amount set for the interferon per standard treatment course in 2014 (CNY 76,000/48 weeks). ^11^ Capitated amount set for DAA treatment per standard treatment course in 2018 (CNY 40,500). ^12^ Six bottles of originator sofosbuvir provided by PAP free of charge for patients in financial hardship as defined by PAP. ^13^ Three bottles of originator sofosbuvir provided by PAP free of charge for low-income patients as defined by PAP. ^14^ Including the first three-month treatment with originator sofosbuvir, which can be reimbursed by insurance following the specialty outpatient care benefit package under the capitated payment program, plus costs for tests & other necessary medications for ADRs for additional free sofosbuvir follow-up treatment provided by APA, which can be reimbursed by the insurance following the common outpatient benefit package. ^15^ Annual average salary per capita of Tianjin employees was applied for the calculation: CNY 67,773 in 2013, CNY 83,428 (2014–2017), CNY 7,073/month in 2018. Obtained from the National Bureau of Statistics of China. http://data.stats.gov.cn; Tianjin Municipal Human Resource and Social Security Bureau. http://hrss.tj.gov.cn/ecdomain/framework/tj/index.jsp (in Chinese, accessed March 31, 2019). ^16^ Annual average disposable income per capita of Tianjin residents was applied for the calculation: CNY 26,359 in 2013, CNY 32,805·11 (2014–2017), CNY 39,506 in 2018. Obtained from the National Bureau of Statistics of China. http://data.stats.gov.cn; Tianjin Statistics Bureau. http://stats.tj.gov.cn/Category_1/Index.aspx (in Chinese, accessed March 31, 2019).

### Capitated provider payment for interferon-based treatment 2014–2017

**Figure 2:**
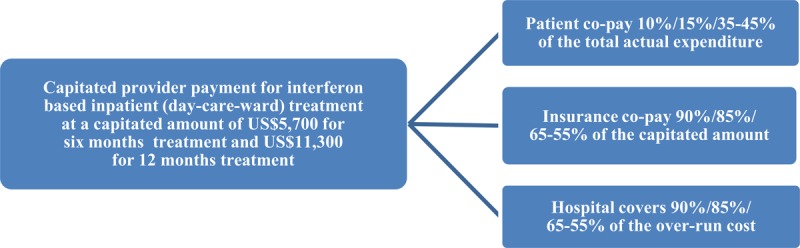
Capitated provider payment for interferon based treatment 2014–2017.

Repeat inpatient care and resource-exhausted FFS provider payments were expensive, and clearly, this approach was not an efficient use of healthcare resources. In 2014, Tianjin Basic Health Insurance shifted the provider payment for hepatitis C treatment from FFS to capitation. The capitated per-patient amount covered expenditures of interferon, ribavirin, as well as necessary tests and ADR treatments was around US$5,700 for six months of treatment and US$11,300 for 12 months of treatment. This amount was co-paid by insurance and patients following the inpatient care benefit package, but patients did not necessarily pay OOP before getting reimbursed [25,26]. Only those who were treated at the day-care-ward, and followed the clinical pathway as defined by Tianjin Basic Health Insurance could register to the pilot program [27], which was implemented in one hepatitis specialty hospital in Tianjin. With this plan, 10% and 15% of the total cost were paid OOP by retirees and employees, and 35–45% by the residents. The OOP patient accounted for 1.1 and 1.6 months of the retirees and employees’ salary, and 9.7–12.5 months of the residents’ disposable income (Figure 2 and Table 1). The designated hospital was co-paid at US$11,300 by insurance and patients with prospective capitated payment method. Hospitals bore the over-run cost and could keep the balance in case that the real treatment cost is less than the capitated amount.

### Capitated provider payment for DAA based treatment with defined clinical pathway since 2018

**Figure 3:**
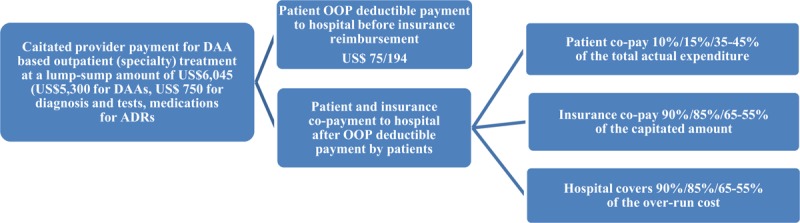
Capitated provider payment for DAA based treatment with defined clinical pathway since 2018. *Notes:* DAA = direct-acting antiviral; OOP=out-of-pocket.

In 2018, when increasing numbers of DAA being registered and marketed in China, Tianjin Basic Health Insurance negotiated the prices of all marketed DAAs. US$5,300 per standard treatment course was agreed for most of DAAs except sofosbuvir/velpatasvir, which was about 60% of the private market price. A fixed amount of US$6,045 was set as per-patient price for outpatient care, valid for two years until 2020. This includes US$750 for necessary diagnosis and tests, medications for ADRs, and monitoring and management fees (including HCV RNA testing but not genotype testing) [21]. Clinical pathways were formulated by Tianjin Basic Health Insurance, which enabled clinicians to provide tailor-made treatment. DAAs included in the clinical pathways are presented in Annex 4. Patients get reimbursed under the specialty outpatient care benefit package. Before getting reimbursed, a deductible of US$75 (residents) and US$194 (employees) have to be paid OOP by patients. With this pilot, 10% and 15% of the total cost is paid OOP by retirees and employees, and 45–55% by the residents. The OOP payment accounts for 0.7 and 1.0 months of the retirees and employees’ salary, and 5.6–6.8 months of the residents’ disposable income (Figure 3 and Table 1). Up until now, three specialty hospitals have been designated, which is co-paid at the amount of US$6,045 by insurance and patients with the prospective capitated payment method. Hospitals bear the over-run cost and keep the balance in case that the real treatment cost is less than the capitated amount. Designated hospitals can directly transfer the prescriptions to the designated retail pharmacies through the health insurance information system; DAAs are dispensed at the retail pharmacies. Five retail pharmacies are designated in Tianjin city.

**Figure 4:**
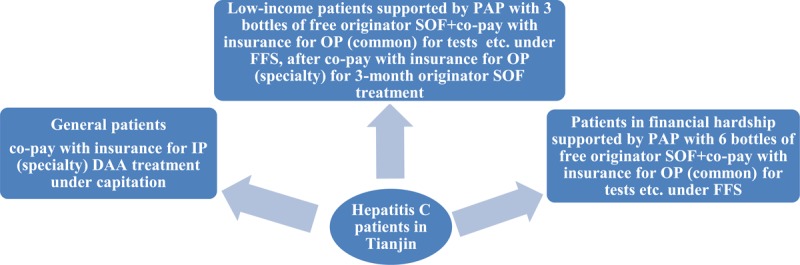
Financing models for DAA treatment of hepatitis C patients in Tianjin since 2018. *Notes:* IP = inpatient; OP = outpatient; PAP = patient assistance program; SOF = sofisbuvir; FFS = fee-for-service.

Patients in financial hardship are eligible for the Patient-Assistance-Program (PAP) created by China Primary Health Care Foundation (CPHCF). They have to be certified by the Tianjin Civil Affairs Bureau and provide a record of receiving minimum living allowance during the past consecutive 12 months. The eligible patients can be provided with up to six bottles of originator sofosbuvir free of charge. Low-income patients (whose immediate family^2^ income was less than 2.5 times the family annual medical expense) can apply up to three bottles of originator sofosbuvir free of charge in case of need more than a three-month treatment. The pre-condition is that patients have to finish in advance three-month treatment with originator sofosbuvir before application, which can be reimbursed by insurance following the capitated specialty outpatient care benefit package. With the PAP, although patients in financial hardship pay nothing to sofosbuvir, they have to pay genotype test OOP and have the expenditures of necessary diagnosis and tests, medications for ADRs co-paid with insurance (following the common outpatient care benefit package). However, the benefit package for common outpatient care is minimal (US$75 OOP payment before getting reimbursed, insurance reimburses 50%, US$450 reimbursement cap). This common outpatient benefit package also applies to the expenditures of continued treatment of low-income patients who require more than three-month treatment. They can register the new insurance coverage to complete their advance treatment, co-pay with insurance under the specialty outpatient benefit package, and have to add genotype testing to their total OOP payment (Figure 4 and Table 1). Hospitals are co-paid by insurance and patients for the actual treatment cost with the retrospective FFS method.

**Figure 5:**
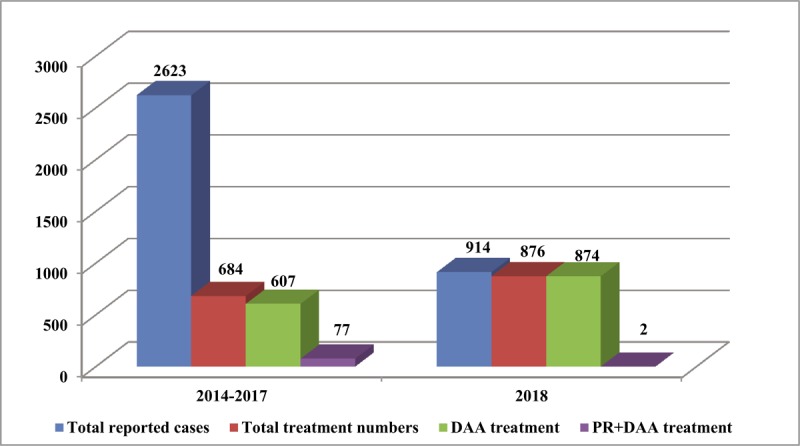
Reported cases and treatment numbers of hepatitis C covered by the Basic Health Insurance programs of Tianjin 2014–2017 vs. 2018. *Notes:* DAA = direct-acting antiviral; PR = pegylated interferon + ribavirin.

By the end of March 2019, there were 876 hepatitis C patients registered the new insurance coverage and treated in Tianjin. Among which, two adopted pegylated interferon + ribavirin + DAA treatment protocol, 874 were treated with DAA (Figure 5). As of the end of March 2019, 153 were cured, 723 were under treatment and follow-up.^3^ There were a total of 148 patients (11 patients in financial hardship and 137 low-income patients) supported by the hepatitis C PAP.^4^

## Discussion

The capitated provider payment mechanism in Tianjin expects to create incentives for the designated hospitals to manage the patient and the health insurance budget in the most cost-effective way, and to achieve tripartite wins: to relieve individual financial burden of patients, to contain costs and to increases efficiency of treatment, and to secure the sustainability of the health insurance. However, within the first year of implementation of the new insurance coverage, there were only 876 hepatitis C patients treated with DAAs in Tianjin. This number is already more than the total number of patients treated during 2014–2017 (684), and it is close to the number of newly reported in 2018 (914). The majority of the former 77 patients under pegylated interferon/ribavirin treatment shifted to DAA treatment (Figure 1); 876 only accounts for 10% of the accumulated notifications during 2004–2018 (8,421) [20]. Considering the notifications before 2004 and the omissions, even adding the patients in financial hardship who received free DAA from PAP (only 11 patients in 2018), and assuming that there will be no new infections in the coming years, Tianjin will have to have at least ten years to treat all the existing patients. This non-optimistic situation calls for further expansion of the treatment program from the existing three hospitals to all tertiary hospitals which establish the department of infectious diseases in Tianjin.

Except for the limited number of pilot healthcare providers, there were other manifold reasons behind the low treatment rate. Financial barriers are the most prominent ones for patient access to highly effective DAA-based hepatitis C treatment. Although the negotiated prices of originator DAAs in Tianjin (US$5,300 per standard treatment course) is less than one-third of the interferon treatment cost before 2014, and it is below the per-patient originator price estimated with lump-sum remuneration by the Australian government in 2017 (US$7,352). It is still several times higher than those obtained by many other middle-income countries. Price of generic sofosbuvir produced by the Brazilian consortia involving private pharmaceutical companies and public laboratories through the PDP program reduced to US$8.50/pill (US$765 per three months) in 2018 [4]. The Brazilian government also negotiated a price of originator sofosbuvir/ledipasvir at US$1,148, and US$1,470 for a pan-genotype originator, sofosbuvir/velpatasvir for a total number of 50,000 treatments in 2019 [28]. The rapid expansion of treatment in Brazil was mostly due to the access to low-priced generics and excellent price reduction of the originator. Brazil’s experience supplied direction for the government of developing countries including China, which is that direct involvement of the central government may secure high political commitment and greater leveraged purchasing power for both the originator and the generics.

With a comparatively high price, the financial burden of patients in Tianjin is still high even under the new insurance coverage, especially for the resident population, whose OOP payments account 45–55% of the total treatment cost. This is much higher than the proportion of the national average of OOP payment to the total health expenditure (30%) [29]. Although patients in financial hardship and low-income patients can get originator sofosbuvir free of charge, it is limited to sofosbuvir only, not covering other DAAs, especially the pan-genotype ones. This means that patients have to pay OOP for genetic testing and other necessary medicines and tests, which will bring an additional financial burden. There was still more spaces for the basic health insurance program of Tianjin to further strengthen its safety-net for the patients in financial hardship.

The second reason might due to that there were very few numbers of patients in financial hardship who benefited from PAP in Tianjin in 2018. The current study shows that the rural population and the unemployed people have the highest incidence of hepatitis C in Tianjin [30], who are most probably categorized as the population in financial hardship. This implies the under-enrollment of the patients in financial hardship to the PAP. Low awareness of patients, weak advocacy of the program, complicated benefit packages, pathway and procedures of the basic health insurance program and transfer to the PAP all might create new barriers of patient access. There is a need to simplify the insurance benefit packages and payment schemes for patients, as well as the procedures for patients to shift from the insurance coverage to the PAP support.

The third reason behind the low treatment might be that alternative approaches of access existed, such as overseas shopping of DAAs. The international price of sofosbuvir already decreased to US$750 for three months treatment in 2017 and has been continuously decreasing. This is much lower than the originator price negotiated by Tianjin (US$5,300). The low-priced DAAs are available in increasing numbers of developing countries like India, Thailand, Argentina, Egypt, Pakistan, and so on [31]. Unless the price is close to the low international level, the overseas shopping of cheaper life-saving medicines may still be an incompetent choice of the dying-to-survive patients. Many studies revealed quality and safety risks to patients [32,33].

The study showed that even in the developing countries, the financial barriers to access to new hepatitis C treatment for patients and society could be unblocked with government commitment and novel financing models. International experiences demonstrated that centralized bulk procurement is a good leverage for price negotiation, mainly when innovative payment approaches were used. To reproduce the initial success of Tianjin, continued efforts are needed for stronger strategic price negotiation, preferably at central level. The case of Tianjin brings implications to the other areas of China and even other developing countries that, government commitment, novel financing model and pooled procurement are critical elements of stronger purchasing power and better secure of treatment.

In December 2019, the government successfully negotiated an 85% reduction of prices for 3 DAAs including sofosbuvir/ledipasvir; sofosbuvir/velpatasvir and elbasvir/grazoprevir compared to retail prices. Health insurance reimbursement for these DAAs started 1 January 2020. Further work will be required to evaluate patient access once provinces begin to implement this policy.

## Additional Files

The additional files for this article can be found as follows:

10.5334/aogh.2763.s1 Annex 1. The standard treatment of hepatitis C recommended by the World Health Organization.

10.5334/aogh.2763.s2 Annex 2. DAAs registered, marketed and under clinical trials in China (by end of March 2019).

10.5334/aogh.2763.s3 Annex 3. List of key informants (sorted by the first letter of the given name).

10.5334/aogh.2763.s4 Annex 4. Clinical pathways defined by Tianjin Health Insurance under capitated provider payment mechanism.

## Footnotes

It is calculated based on the lowest (CNY 11,200/month) and highest (CNY 19,660/month) market price of originator sofosbuvir in China, obtained from the key informants’ interview of the hepatitis C Patient Assistance Program in China. Conversions between US$ and CNY were made with a rate of 1 US$ = 6.7 CNY. This rate was applied throughout this manuscript.

Defined by the Patient Assistance Program as family members including parents, children, husband or wife.

Data were extracted from the database of the Tianjin Healthcare Security Administration and Tianjin Healthcare Security Management and Settlement Center.

Data were extracted from the database of the Patient Assistance Programme managed by the China Primary Health Care Foundation.
