# Supplementary material for: Unblocking Barriers of Access to Hepatitis C Treatment in China: Lessons Learned from Tianjin
Source: Ann Glob Health. 2020 Apr 6;86(1):36. doi: 10.5334/aogh.2763 (PMC7181951; doi:10.5334/aogh.2763)
Supplement: Annex 1. — The standard treatment of hepatitis C recommended by the World Health Organization. [file agh-86-1-2763-s1.pdf]

Annex 1 The standard treatment of hepatitis C recommended by the World Health Organization

A new infection with HCV does not always require treatment, as the immune response in some people will clear the infection. However, when HCV infection becomes chronic, treatment is necessary. The goal of hepatitis C treatment is cure.

WHO's updated 2018 guidelines recommend therapy with pan-genotypic direct-acting antivirals (DAAs). DAAs can cure most persons with HCV infection, and treatment duration is short (usually 12 to 24 weeks), depending on the absence or presence of cirrhosis.

WHO recommends treating all persons with chronic HCV infection over the age of 12. Pan-genotypic DAAs remain expensive in many high- and upper-middle-income countries. However, prices have dropped dramatically in many countries (primarily low-income and lower middle-income countries), due to the introduction of generic versions of these medicines.

Access to HCV treatment is improving but remains too limited. In 2017, of the 71 million persons living with HCV infection globally, an estimated 19% (13.1 million) knew their diagnosis, and of those diagnosed with chronic HCV infection, around 5 million persons had been treated with DAAs by the end of 2017. Much more needs to be done in order for the world to achieve the 80% HCV treatment target by 2030.

Source: The World Health Organization. Key facts about Hepatitis C. (posted on 9 July 2019). Available from: https://www.who.int/news-room/fact-sheets/detail/hepatitis-c. Accessed 7 February 2020.
