# Supplementary material for: Unblocking Barriers of Access to Hepatitis C Treatment in China: Lessons Learned from Tianjin
Source: Ann Glob Health. 2020 Apr 6;86(1):36. doi: 10.5334/aogh.2763 (PMC7181951; doi:10.5334/aogh.2763)
Supplement: Annex 2. — DAAs registered, marketed and under clinical trials in China (by end of March 2019). [file agh-86-1-2763-s2.pdf]

Annex 2 DAAs registered, marketed and under clinical trials in China (by end of March 2019)

Company	Genotype	NS3/4A	NS5A	NS5B
BMS	GT1b	asunaprevir	daclatasvir	
Gilead	GT1,2,3,6	/	/	sofosbuvir
	GT1-6	/	sofosbuvir/velpatasvir	
	GT1,4,5,6	/	sofosbuvir/ledipasvir	
Abbvie	GT1	paritaprevir/ombitasvir/ritonavir		dasabuvir
MSD	GT1,4	grazoprevir/elbasvir		/
Ascletis (Local)	GT1	danoprevir	/	/
J&J	GT1	simeprevir (registered not marketed)	/	/
Abbvie	GT1-6	glecaprevir/pibrentasvir		/
Pharco	GT4	ravidasvir/sofosbuvir		/
Gilead	GT1-6	sofosbuvir/velvelasvir/voxilaprevir		
