# Supplementary material for: Unblocking Barriers of Access to Hepatitis C Treatment in China: Lessons Learned from Tianjin
Source: Ann Glob Health. 2020 Apr 6;86(1):36. doi: 10.5334/aogh.2763 (PMC7181951; doi:10.5334/aogh.2763)
Supplement: Annex 3. — List of key informants (sorted by the first letter of the given name). [file agh-86-1-2763-s3.pdf]

Annex 3 List of key informants (sorted by the first letter of the given name)

1. Bin ZHAO, Research Fellow, China Social Security Research Institute of National Bureau of Healthcare Security Administration
2. Chengzhen LU, Chief physician, Tianjin No. 2 People's Hospital
3. Dachuan LI, Director of Division, Bureau of Medical Administration, National Health Commission
4. Dongmei XIA, Director of Tianjin program, China Primary Healthcare Foundation
5. Guowei DING, Chief Physician, National Center for AIDS/STD Control and Prevention, Chinese Center for Disease Control and Prevention
6. Hong MAO, Deputy Director General, Tianjin Healthcare Security Administration
7. Hong Ren, Division of Prevention and Control of Hepatitis, Shanghai Center for Disease Control and Prevention
8. Hui ZHUANG, Academician of the Chinese Academy of Engineering, Professor of Pathogen Biology, Medical Science Center of Peking University
9. Jiaqi KANG, Director, China Hepatitis Prevention and Treatment Foundation
10. Jidong JIA, Professor of Medicine, Director of Liver Research Center, Beijing Friendship Hospital, Capital Medial University
11. Jing LIANG, Physician, Tianjin No.3 Central Hospital
12. Jinyan HAN, Director of Division of Disease Prevention and Control, Tianjin Health Commission
13. Lai WEI, Director, Hepatitis Disease Research Institute, People's Hospital of Peking University
14. Lijie CHEN, Principal Staff, Zhejiang Healthcare Security Administration
15. Lin PAN, Senior Researcher, National Center for AIDS/STD Control and Prevention, Chinese Center for Disease Control and Prevention
16. Mingyang LI, Director, China Healthy Liver Promotion Center
17. Sen WANG, Principal Staff, Tianjin Health Commission
18. Tao ZHANG， Principal Staff, Tianjin Healthcare Security Administration
19. Xiangjie LI, Director, China Primary Healthcare Foundation
20. Xiaohua HUANG, Project Manager, Ascletis Pharma Inc.
21. Xiaojin SUN, Research Assistant, National Immunization Program Chinese Center for Disease Control and Prevention
22. Xin CHEN, Physician, Tianjin No.1 People's Hospital
23. Yoke Ling CHEE, Director of Research, Third World Network
24. Yunhong SUN, Chief Nurse, Tianjin No. 2 People's Hospital
25. Zhenyan ZHU, Project Manager, Beijing Representative Office of the Heinrich-Böll-Stiftung
