# Supplementary material for: Unblocking Barriers of Access to Hepatitis C Treatment in China: Lessons Learned from Tianjin
Source: Ann Glob Health. 2020 Apr 6;86(1):36. doi: 10.5334/aogh.2763 (PMC7181951; doi:10.5334/aogh.2763)
Supplement: Annex 4. — Clinical pathways defined by Tianjin Health Insurance under capitated provider payment mechanism. [file agh-86-1-2763-s4.pdf]

Annex 4 Clinical pathways defined by Tianjin Health Insurance under capitated provider payment mechanism

Medicines	Genotype	Treatment course (weeks)	Other medications	Test
asunaprevir/daclatasvir	I b	24	ribavirin+ADRs	Every 2-4 weeks
sofosbuvir/daclatasvir	1-6	12	ADRs	Every 2-4 weeks
Sofosbuvir/ pegylated interferon/ribavirin	I b	12	ADRs	Every 1-2 weeks
ombitasvir/paritaprevir/ritonavir/dasabuvir	I a/ I b	12	ADRs	Every 1-2 weeks
sofosbuvir/ribavirin	2/1、3、6	12/24	ADRs	Every 1-2 weeks
danoprevir/ ritonavir/pegylated interferon/ribavirin	I b	12	ADRs	Every 1-2 weeks
sofosbuvir/velpatasvir	1-6	12	ADRs	Every 2-4 weeks
grazoprevir/elbasvir	1/4	12	ADRs	Every 2-4 weeks
